# Supplementary material for: Cell Tree Rings: the structure of somatic evolution as a human aging timer
Source: GeroScience. 2024 Jan 4;46(3):3005–19. doi: 10.1007/s11357-023-01053-4 (PMC11009167; doi:10.1007/s11357-023-01053-4)
Supplement: Supplementary file 1 — (PDF 592 kb) [file 11357_2023_1053_MOESM1_ESM.pdf]

Supplementary Information: Tree Metrics

Cell Tree Rings: the structure of somatic evolution as a human aging timer

Attila Csordas[1,2], Botond Sipos[1], Terezia Kurucova[3,4], Andrea Volfova[5], Frantisek Zamola[5], Boris Tichy[3], Damien G Hicks[1,6]

[1] AgeCurve Limited, Cambridge, CB2 1SD, UK
[2] Doctoral School of Clinical Medicine, University of Szeged, Szeged, H–6720, HU
[3] CEITEC – Central European Institute of Technology, Masaryk University, 62500 Brno, CZ
[4]Department of Experimental Biology, Faculty of Science, Masaryk University, 62500, Brno, CZ
[5] HealthyLongevity.clinic Inc, 540 University Ave, Palo Alto, CA 94301, US
[6] Swinburne University of Technology, Hawthorn, VIC 3122, AU

Corresponding author

Attila Csordas, email: attila@agecurve.co.uk

Cell Tree Metrics

The features are split into 5 groups based on their technical properties.

Group I. Spectral tree metrics

These features implement wavelet [1] or spectral graph [2] analysis of tree shape. In wavelet analysis, the tree is regarded as a signal on the domain of a complete tree and analysed using Haar wavelets. In spectral graph analysis, the eigenvalues of the graph Laplacian are calculated.

I/a. Wavelet-based features

In [1] a generalized Fourier transform for functions on a complete tree was derived from symmetry requirements and found to be related to the Haar wavelet basis. Here we apply this transform to the structure of the tree itself. We do this by representing the tree on the nodes of a complete tree: if the tree exists at a node of the complete tree the signal is 1; if it is absent the signal is 0. Since the tree is a small subset of the complete tree there are many zeros in the

signal. Thus, to save memory, the transformation is performed by summing only the elements in the transform matrix that multiply 1's in the signal.

The result is a spectrum that characterises the strength of bifurcations in the tree as a function of ℓ, the generation in which the bifurcation originates. This spectrum is summarized using the following metrics:

- avgen_10: The sum of coefficients up to ℓ =10.
- avgen_20: The sum of coefficients up to ℓ =20.
- avgen_40: The sum of coefficients up to ℓ =40.
- avgen_60: The sum of coefficients up to ℓ =60.
- avgen_80: The sum of coefficients up to ℓ =80.
- avgen: The sum of all coefficients.
- avgen_half_g_max: The sum of all coefficients normalized by half the depth of the tree.

I/b. Graph Laplacian tree features

Features in this category are based on either the graph Laplacian, which considers the branching order itself but ignores branch lengths, or the modified graph Laplacian [2], which incorporates branch lengths as well.

Features based on the Graph Laplacian (GL)

Here the tree is represented as a graph with branches treated as edges and the internal nodes and leaves treated as vertices. Following standard convention, the graph Laplacian is calculated from L = D - A, where D is the diagonal degree matrix and A is the adjacency matrix, composed of a 1 if a pair of nodes is adjacent, and a 0 if not. The vector of eigenvalues of the graph Laplacian is called the spectrum [Lewitus & Morlon 2016]. The spectrum is characterised using several features:

- Algebraic Connectivity: the second smallest eigenvalue of the non-modified GL matrix.
- Wiener index: 0.5 times the sum of the shortest-path distances between each pair of reachable nodes.
- 'hic': the number of eigenvalues less than or equal to 1.0
- 'cso1': the number of eigenvalues greater than 1.0

Features based on the Modified Graph Laplacian (MGL)

Here the tree is represented as a graph where all the internal nodes and external leaves are used as nodes. The graph Laplacian is modified to account for the length of the branches. This is

done by giving each term in the adjacency matrix a weighting determined by the distance between the associated node pair. Eigenvalues (except zero) are log-transformed by taking their natural log. Gaussian kernel convolution is used to also produce a continuous Spectral Density Profile (SDP).

The following eight tree features are determined from the resulting MGL spectrum:

Kurtosis: the fourth central moment divided by the square of the variance of the eigenvalues of the MGL.

Tracer: the maximum height of the Spectral Density Profile.

Skewness: the skewness statistics of the Spectral Density Profile calculated from the 3rd moment μ_3 and 2nd moment μ_2 of the distribution of the eigenvalues of the MGL as $\mu_3/\mu_2^{3/2}$.

Tree Imbalance: the product of Kurtosis and Skewness.

Modified Maximum Eigenvalue: the largest eigenvalue of the MGL.

Modified Maximum Eigengap: the largest difference between two consecutive eigenvalues of the MGL.

Modified Algebraic Connectivity (mAC): the second smallest eigenvalue of the MGL.

Mode Value: The most frequent eigenvalue of the spectral density profile.

Group II. Phylogenetic Branch Length Features and derivatives

Branch Length Log Norm

- The sum of all the branch lengths is computed using the ape R package.
- The sum is normalized by the natural logarithm of the number of tips of the tree.

Mean Branch Length

- The sum of all the branch lengths in the tree is computed using the Bio.Phylo python module.
- The mean branch length is computed by dividing the sum of all the branch lengths by the number of all nodes in the tree.

Entropy based features

These are measures of the spread of pairwise distances between nodes on the tree. The nodes involved can be just the tip nodes or they can be both internal and tip nodes.

Entropy Tips: This requires first finding a histogram of all the pairwise distances between tips of the cell lineage tree, placing each into 50 bins. The Shannon entropy is then calculated from the histogram.

Entropy All Nodes: This is the same as Entropy Tips except pairwise distances between all internal and tip nodes are used.

Group III. Traditional Phylogenetic Features

The tree features listed here are standard in the phylogenetics literature.

Colless index: A statistic designed to assess tree symmetry this is a recursive sum of the differences between left and right leaves at every stage of the tree. Does not consider branch lengths.

Sackin index: A statistic designed to assess tree symmetry this is a sum of all the branches between a root and a leaf summed up for all leaves. Does not consider branch lengths.

Cherries: The number of pairs of adjacent tips on a tree.

Total cophenetic index: Sum of the branch lengths of the lowest common ancestors for all pairs of leaves in the tree. Normalised by the number of leaves in the tree.

tipRootPatr: Captures both topology and branch length information. Each tip node is a certain distance from the root. This distance is summed across all tips.

Group IV. Distance Matrix based Branch Length Features

The features in this group were specifically adapted to the cell lineage tree aging application.

tipDistNorm:

- Distances between the tips of the tree are computed using the branch length information with the distTips function from the adephylo package.
- The sum of all the distances between the tips is computed.
- The sum is normalized by the square of the number of tips used to generate the tree.

tipDistLogNorm:

- Distances between the tips of the tree are computed using the branch length information with the distTips function from the adephylo package.
- The sum of all the distances between the tips is computed.
- The sum is normalized by the natural logarithm of the number of tips in the tree.

tipDistSD

- Distances between the tips of the tree are computed using the branch length information with the distTips function from the adephylo package.
- The standard deviance of the distances between the tips is calculated.

Group V. PowerGraph Features

The square of a binary cell lineage tree is a powergraph and is found by defining node v and node u to be adjacent if, in the original tree, u and v are at most two edges away from each other. Once this powergraph has been generated the Graph Laplacian Matrix can be generated as described above. The natural logarithm of the spectrum of eigenvalues of the Graph Laplacian is computed and the algebraic connectivity found from the second smallest eigenvalue. The algebraic connectivity of a powergraph is a well-known feature of graph robustness [3].

AC_2: This is the algebraic connectivity from the graph Laplacian of the powergraph.

mAC_2: This is the algebraic connectivity from the modified graph Laplacian of the powergraph.

Cell Tree Age model with feature pre-selection using UPGMA

Applying feature pre-selection (*Regression Analysis*) on the HLC training set resulted in the best performing Cell Tree Age model using 5 tree features 'tipDistNorm', 'mMax_eigengap', 'mMaxEigen', 'AC_2', 'tipRootPatr' (see definition in Cell Tree Metrics section above) and interaction terms with ‘Sex’ binary variable with the smallest errors and highest correlation coefficients: r=0.908, p = 0.000001, R^2=0.825, MAE=5.690, MdAE=3.480, RMSE=7.782. Supplementary Figures 1A and 1B show how the performance metrics change when using between 1 to 32 of the ranked features. Results from the best performing model, with 6 features, are shown.

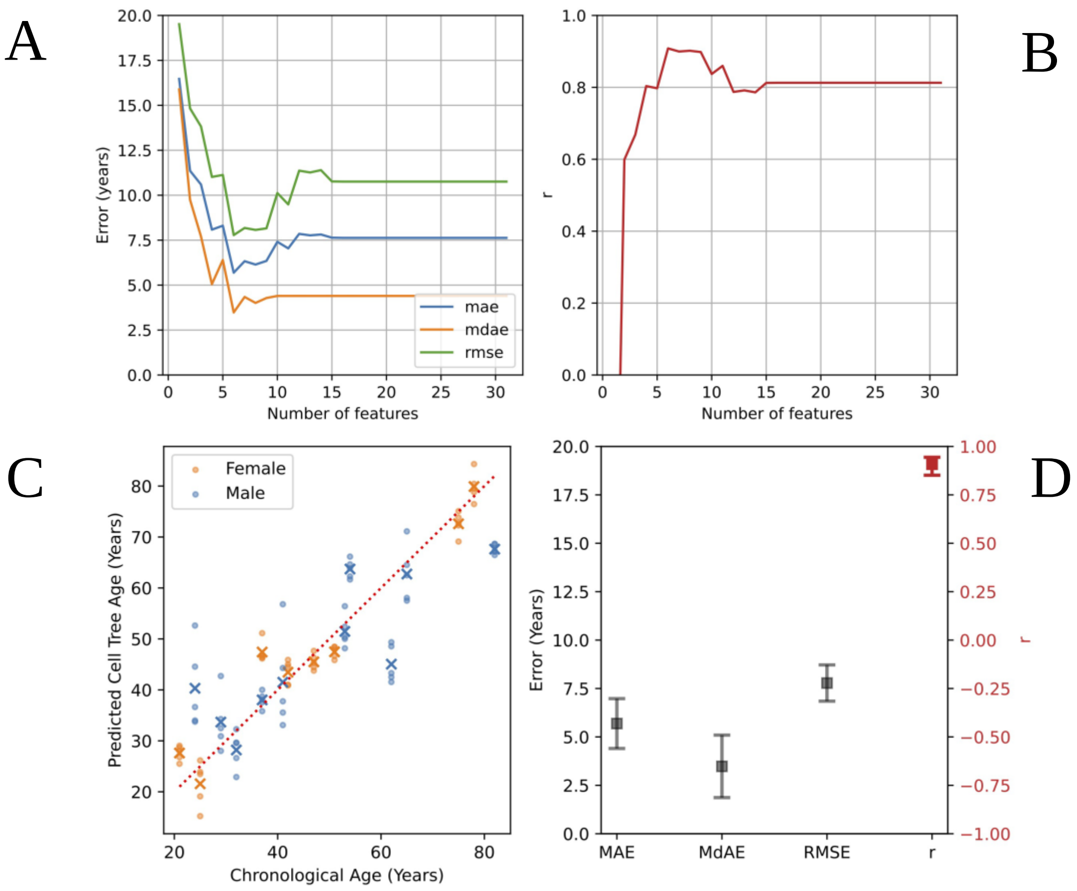

Supplementary Figure 1: Panel A and B: Performance metrics as a function of the number of the top ranked features used. The most accurate model built using UPGMA trees is the 6-feature model (5 tree metrics and Sex). Panel C and D: Cell Tree Age performance on 18 human HLC blood samples using the 6 feature model selected with feature pre-selection.

Cell Tree Age performance on public data with feature pre-selection using UPGMA

Supplementary Figure 2 shows performance of the Cell Tree Age model on the 18 AIDA datasets with only 4 tree features selected with 'Sex' as interaction term. These 4 tree features are 'tipDistNorm', 'mMax_eigengap', 'mMaxEigen', 'AC_2' and the performance metrics are r=0.721, p = 0.00074, R^2=0.519, MAE=8.942, MdAE=7.548, RMSE=10.894.

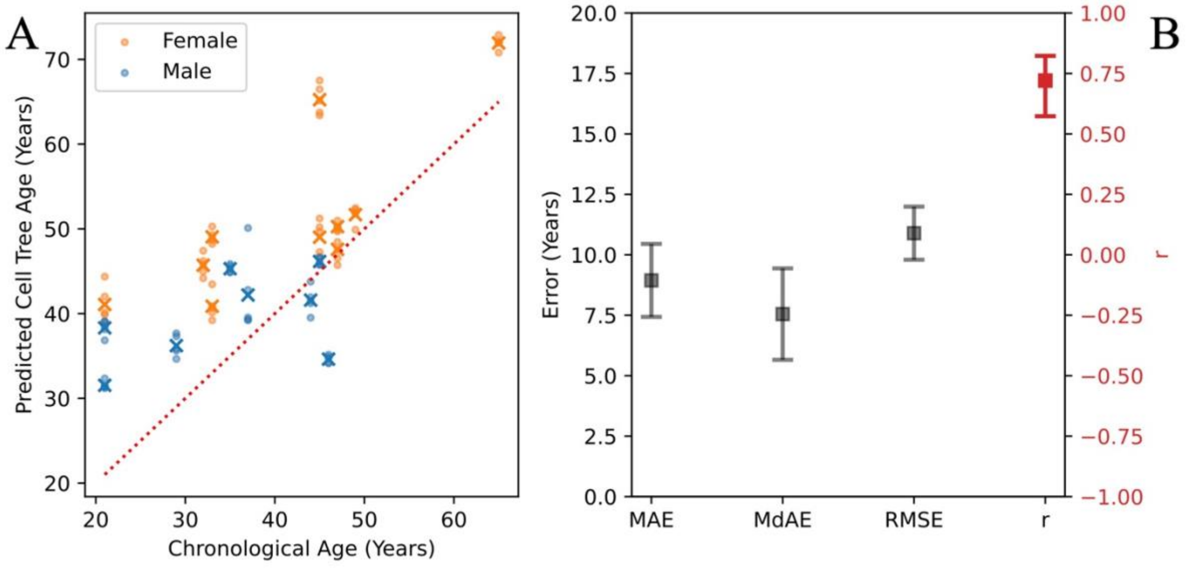

Supplementary Figure 2: Panel A and B: Performance of the model on the public AIDA dataset, after training on the HLC dataset. Uses feature pre-selection with 4 features plus Sex. Panel B: the statistics of leave-one-out cross validation shows mean performance metrics of the model, MAE, MdAE, RMSE are Mean Absolute Error, Median Absolute Error, Root Mean Squared Error in years, correspondingly, and r is Pearson's Correlation Coefficient.

Cell Tree Age model and feature pre-selection with Maximum Likelihood

For evaluating the Maximum Likelihood tree method, 5 pseudo-replicate trees using 700 randomly sampled cell each were constructed with IQ-TREE. Supplementary Figure 3 below shows the default all feature model with performance metrics r=0.694, p = 0.00139, R^2 =0.482, MAE=9.701, MdAE=6.060, RMSE=13.424.

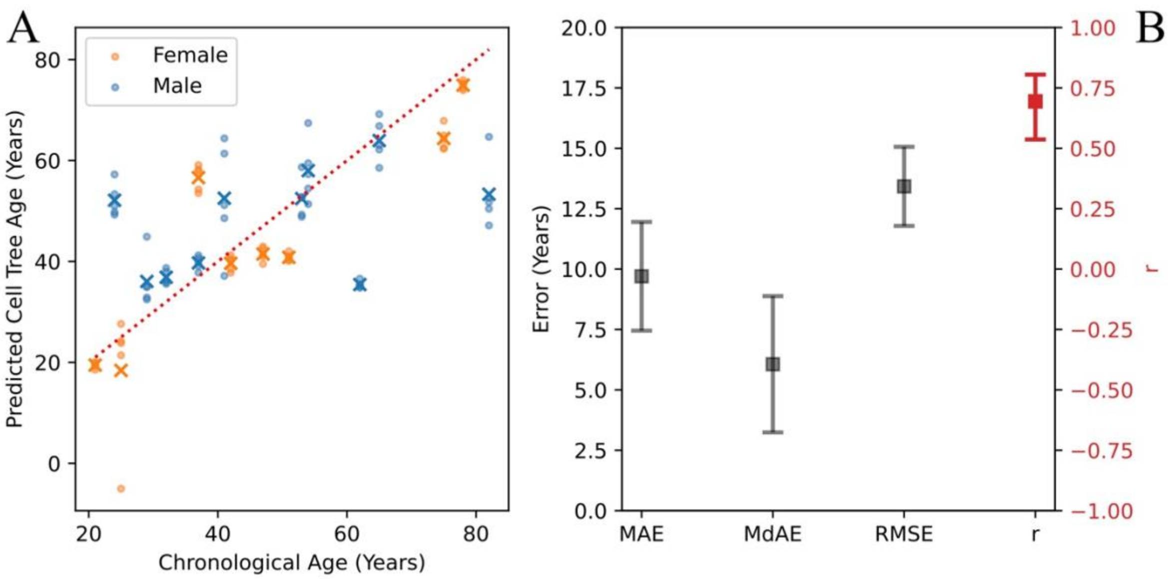

Supplementary Figure 3: Cell Tree Age prediction performance on the 18 HLC human blood samples based on trees inferred using maximum likelihood (rather than UPGMA). This model uses only three tree features and Sex as an interaction term.

Supplementary Figure 4 below shows performance metrics of scanning through the number of ranked features with feature pre-selection and the scatter plot and performance metrics of the best performing maximum likelihood-based model with performance metrics 6 features selected with performance metrics r=0.797, p = 0.00007, R^2=0.636, mae=8.260, mdae=5.654, rmse=11.144. The 5 tree features are 'mMaxEigen', 'mMax_eigengap', 'avgen_10', 'avgen', 'tipRootNodes' plus 'Sex', with ‘Sex-only’ interaction terms allowed.

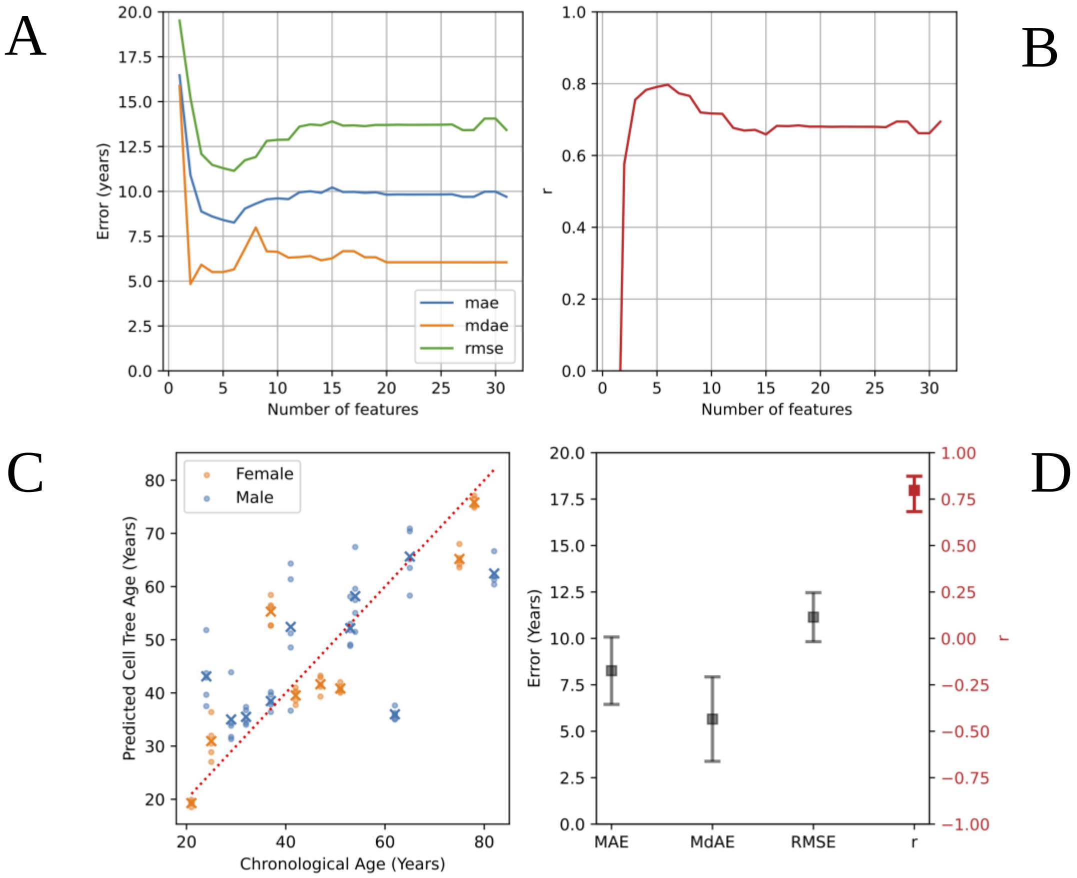

Supplementary Figure 4: Panel A and B: Feature pre-selection showing performance metric changes when selecting an increasing number of minimum features, error terms on A and correlation coefficient on the right panel. The most accurate model built using Maximum Likelihood trees is a 6 feature model (5 tree metrics and Sex), where mean and root mean squared errors are the smallest and correlation coefficient is the highest. Panel C and D: Performance of the most accurate 6 feature model selected with feature pre-selection.

References

1. Hicks DG, Speed TP, Yassin M, Russell SM. Maps of variability in cell lineage trees. PLoS Comput Biol. 2019 Feb 12;15(2):e1006745. doi: 10.1371/journal.pcbi.1006745.
2. Lewitus E, Morlon H. Characterizing and Comparing Phylogenies from their Laplacian Spectrum. Syst Biol. 2016 May;65(3):495-507. doi: 10.1093/sysbio/syv116.
3. Jamakovic A, Uhlig S. On the relationship between the algebraic connectivity and graph's robustness to node and link failures, in: 2007 Next Generation Internet Networks.
